# Experimental and Numerical Investigations on Seismic Behavior of RC Beam to PVC-CFRP Confined Concrete Column Exterior Joint with Steel Tube Connector

**DOI:** 10.3390/polym14214712

**Published:** 2022-11-03

**Authors:** Siyong Tan, Feng Yu, Haiying Bao, Yucong Guan

**Affiliations:** 1 Department of Civil Engineering and Architecture, Anhui University of Technology, Maanshan 243099, China; 2 Shanghai Baoye Group Corp., Ltd., Shanghai 200941, China

**Keywords:** PVC-FRP confined concrete, joint, beam, column, concrete filled steel tube, seismic behavior

## Abstract

Recently, substantial investigations were developed on a polyvinyl chloride (PVC)-carbon fiber-reinforced polymer (CFRP) confined concrete (PFCC) structure owing to its superior mechanical behavior and durability. However, a convenient and effective joint configuration between the PFCC columns and reinforced concrete (RC) beams still requires in-depth study. In the present work, the seismic performance of an RC beam to PFCC column exterior joint with steel tube connector (STC) is systematically studied. Eleven joint specimens are fabricated and tested, with the steel ratio of STC, reinforcement ratio of the frame beam, axial compression ratio, stirrup ratio of the joint and CFRP strips spacing as the design parameters. The experimental results, that is, 8 the failure modes, hysteretic response, ductility, strength, stiffness and energy dissipation, are analyzed. All specimens exhibit joint shear failure, although the joints with STC exhibit significantly better performance those of ordinary joint. In addition to reducing the axial compression ratio, the reinforcement ratio of the frame beam or increasing the stirrup ratio of the joint can also produce a positive effect. Furthermore, the numerical analysis of the exterior joints is performed; the calculated skeleton curves agree well with the test results, and additional parametric studies (i.e., the diameter, height and concrete strength of the joint) are carried out based on the verified numerical model.

## 1. Introduction

Previous studies have proven that concrete-filled tube (CFT) systems can significantly enhance the mechanical properties and durability of encased concrete; CFT has emerged as one of the essential strengthening forms for high-rise and pile foundations [1,2]. In particular, concrete-filled steel tube (CFST) and concrete-filled fiber-reinforced polymer (FRP) tube (CFFT) columns, as two of the commonest forms of CFT structures, have been extensively studied in the component and system levels [3,4,5].

However, owing to the corrosion of steel tubes, as well as the brittle fracture and high price of FRP materials [6,7], several investigations have turned to novel types of confining tubes [8,9,10]. In view of the considerable corrosion resistance of polyvinyl chloride (PVC) and excellent confinement effect of FRP strips, the PVC-CFRP confined concrete (PFCC) column was introduced [11]. So far, the mechanical properties of the PFCC column have been extensively studied, such as its static, seismic and long-term behavior, which exhibits the advantages of lightweight, high bearing capacity, excellent ductility and durability [12,13,14].

It is well recognized that the connection between the CFT columns and reinforced concrete (RC) beams is an imperative research field for CFT systems [15,16,17]. Currently, studies involving the connection between the CFT columns and RC beams are mainly focused on the CFST structure [18,19,20,21,22,23]. Among them, according to whether the steel tube is interrupted in the joint core, the joints can be divided into column-through system and beam-through system. For the former, although the expected joint stiffness can be measured, the following defects are commonly demonstrated: (1) The transmission of joint shear force relies on external steel members, such as reinforced loop plate [24], steel corbels [25,26] and plate-studs [27], which may result in stress concentration, whereby (2) the mentioned steel members are required to be bolted or welded to the CFST columns on-site, which complicates the process of construction.

For the beam-through system, the longitudinal bars of the RC beam can penetrate through the joint core directly, which significantly improves the integrity of the joint area. However, considering the discontinuity of the steel tube may weaken the confinement on the joint core concrete, a novel joint form (i.e., a steel tube connector (STC) with the surrounded ring stirrups is employed in the joint core to confine the core concrete) is proposed [28]. Wang et al [29] carried out the axial compression experiments on the RC beam to CFST column joint with STC. The results manifested that STC could effectively transmit the vertical loads and exhibited excellent bearing capacity and deformability. Subsequently, Fu [30] developed a finite element (FE) analysis on the composite joints, and the method for calculating the compressive strength was proposed. The hysteretic behavior of composite joints was experimentally and numerically investigated by dozens of scaled joints [31,32]. The results demonstrated that all specimens were damaged with the occurrence of the plastic hinge, the full hysteretic curves reflected that the joints presented superior energy dissipation capacity and ductility.

As mentioned above, multiple studies have confirmed the feasibility of the joint with STC. Similar measures were applied to deal with connections between the PFCC columns and RC beams, and a series of static experiments and FE simulations were performed [33,34,35,36]. The results revealed that the bearing capacity, deformability and stiffness of the specimens increased considerably with the improvement in the steel ratio of STC. Moreover, the prediction formula of axial bearing capacity and the related structural measures were proposed.

Nevertheless, given the vulnerable portions in the earthquakes, it is far from sufficient to only conduct the investigations on the static performance of the developed joints [37,38]. Meanwhile, the exterior joints are only confined by the unilateral frame beam, resulting in its damage in the earthquake are more serious than the interior joints. Therefore, ten RC beam to PFCC column exterior joints with STC and one ordinary exterior joint are prepared and tested under low cycle loading in the work. The impacts of the steel ratio of STC, reinforcement ratio of the frame beam, axial compression ratio, stirrup ratio of the joint and CFRP strips spacing on the seismic performances are systematically analyzed. Furthermore, the numerical analysis of the joint is established utilizing the OpenSees and verified by the experimental data. Finally, the additional parametric studies are conducted and some configuration suggestions are proposed, which is expected to provide a theoretical guideline for the application of the PFCC structures.

## 2. Test Preparation

### 2.1. Design of the Joints

**Figure 1:**
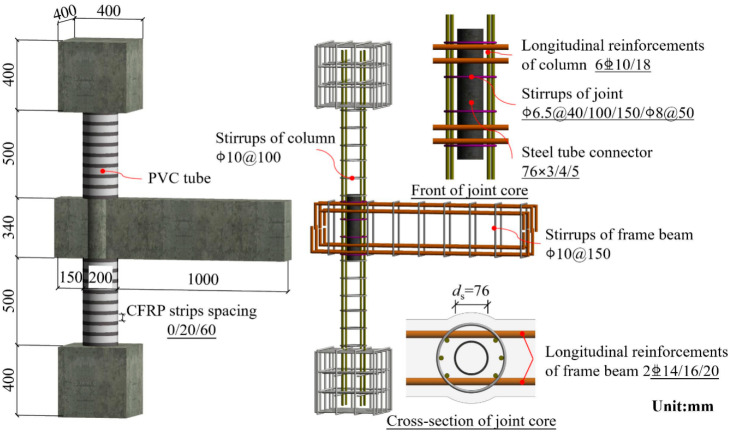
The overall appearance and detailed reinforcements of the specimens.

To ensure that the loading conditions of the specimens were in accordance with the stress characteristics of the frame joints, a T-shaped joint consisting of the upper and lower columns and a unilateral frame beam were intercepted as the investigation objects. The overall appearance and detailed reinforcements of the specimens are plotted in Figure 1.

A total of ten RC beam to PFCC column exterior joints with STC and one reference joint were manufactured. The design principle of “strong members, weak joint” was adopted because the main purposes of this experiment was to investigate the failure modes and seismic behavior of the composite joints. The impacts of the steel ratio of STC $\alpha_{\mathrm{st}}$, reinforcement ratio of the frame beam $\rho_{b}$, axial compression ratio n, stirrup ratio of the joint $\rho_{v}$ and CFRP strips spacing $s_{f}$ are considered. The detailed parameters are depicted in Table 1.

Additionally, the thickness of the PVC tubes adopted for the specimens was 7.8 mm and the outer diameter was 200 mm. Two layers of 0.111 mm thick CFRP strips are wrapped around the PVC tubes in the hoop direction, and the width of each layer is 20 mm. It is worth mentioning that an additional layer of CFRP strip is utilized at the PFCC column ends to prevent its premature failure. The stirrups of the PFCC columns and frame beams for all specimens are A10@150 and A10@100, respectively.

### 2.2. Material Properties

According to GB/T 50152-2012 [39], the cube specimens with a side length of 150 mm are prepared when pouring the joint specimens, the average compressive strength and elastic modulus of the concrete obtained from the cube test are 22.28 Mpa and 3.06 × 10^4^ Mpa, respectively. The main index properties of the steel material, PVC tubes and CFRP strips adopted in the test are measured according to GB/T 228.1-2010 [40], GB/T 8804.1-2003 [41] and GB/T 3354-2014 [42], respectively, and the measurement results are recorded in Table 2.

### 2.3. Loading Device and Protocol

**Figure 2:**
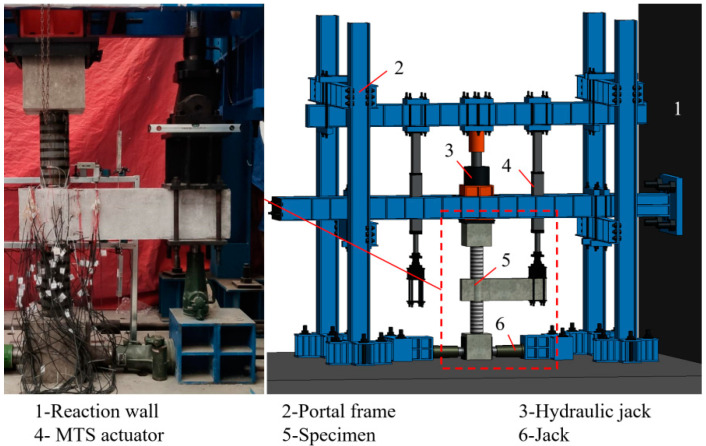
The loading and measurement device.

As presented in Figure 2, the vertical load transmitted from the superstructure is performed by the hydraulic jack on the column top, the MTS actuator attached to the portal frame provides the cyclic loading to simulate the seismic action. The data acquisition system automatically captures the vertical loads and displacements at the frame beam end from the transducers inside the MTS actuator.

**Figure 3:**
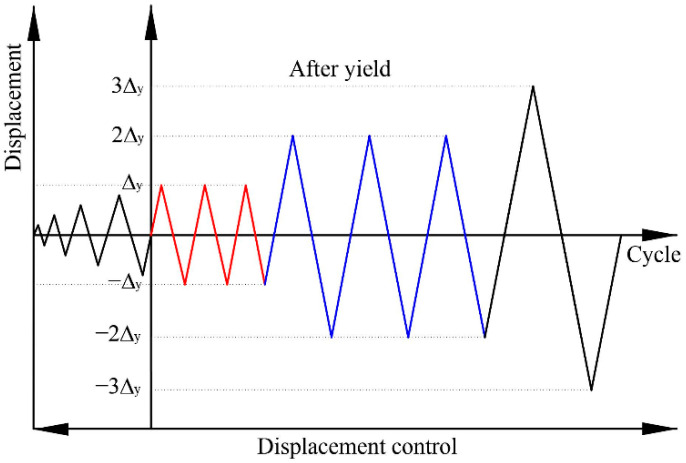
The loading protocol.

Referring to the test method specified in China Industry Standard JGJ/T 101-2015 [43], the displacement control is adopted, as illustrated in Figure 3. The loading process can be divided into two stages: Before the specimen yield, the load cycles for each control level performs once, and the displacement amplitude increases by 1 mm after each cycle. Then, the load for each control level repeats thrice with an integral multiple yield displacement Δ_y_ as the displacement amplitude until the specimen is destroyed or the load declines to 85% of the peak bearing capacity. However, considering the relatively poor deformability in the joint core of the designed specimens, the method of identifying the yield displacement Δ_y_ by the reinforcements yielding may result in the premature failure of the specimens. Consequently, in accordance with the crack development and reinforcements strain of the specimens in the pre-test, 6 mm is selected as the yield displacement in subsequent test. Nevertheless, regarding the subsequent seismic performance analysis, the veritable yield displacement is determined by the energy equivalence method [44] based on the obtained load-displacement skeleton curves.

## 3. Experimental Observations and Results

**Figure 4:**
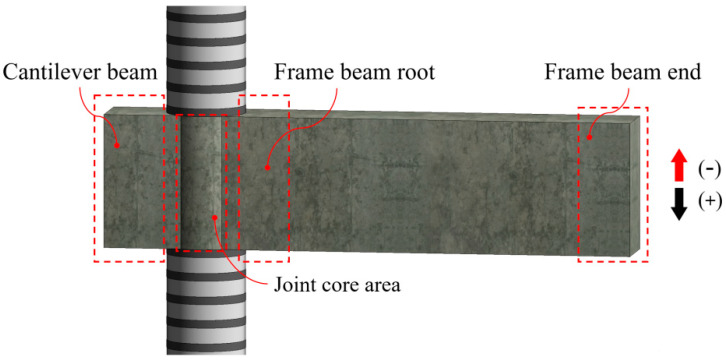
Denomination of the components.

To facilitate the description of the experimental phenomenon, the components of the joints are denominated as shown in Figure 4.

### 3.1. Crack and Failure Modes

**Figure 5:**
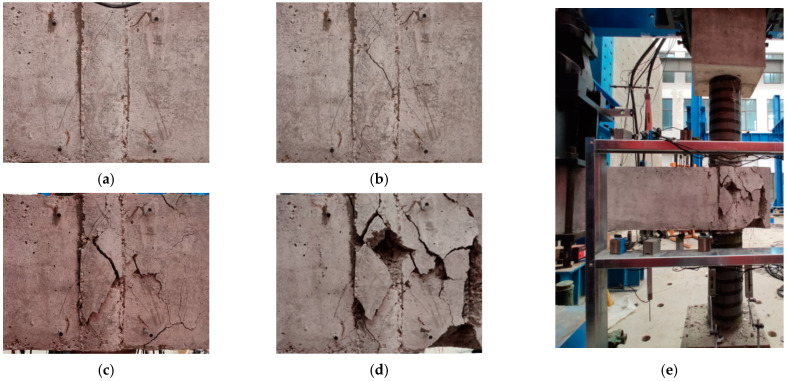
Typical crack and failure mode. (**a**) Oblique cracks. (**b**) Crossed oblique cracks. (**c**) Cracks extending. (**d**) Concrete spalling. (**e**) Overall failure mode.

As expected, the shear failure of the joint core area is observed in all specimens, and the variation in the structural parameters has no apparent impact on the final failure mode. Meanwhile, the specimens exhibit similar crack mode except for an insignificant variance in the timing of crack appearance. The typical crack and failure mode are depicted in Figure 5, and the exhaustive description of the failure process is developed as follows.

At the initial loading stage, the specimens have no obvious cracks and exhibit elastic response. With the improvement of the displacement in the frame beam end (hereinafter called displacement), several oblique cracks emerge in the joint core area. Compared with the specimens PJ-2 and PJ-4, the above-mentioned oblique cracks of the specimen PJ-5 is accompanied with a higher displacement, which is mainly because the joint stirrups can provide a slight confinement for the surrounding concrete. At the displacement of 2Δ_y_, the oblique cracks are developed into the crossed oblique cracks and extends to the frame beam root and the cantilever beam. Until the peak bearing capacity is reached, the cracks present a stable development without new cracks occur, only the increase in the crack width. In this stage, the improvement in axial compression ratio can further accelerate the cracks development. When loading to the cycle with the displacement of 6Δ_y_, severe concrete spalling is exhibited in the joint core area, an inevitable bond-slip between the STC and the joint concrete is observed, which indicates that the experiment is accomplished. There is no significant damage in the PFCC column during the whole loading process.

### 3.2. Hysteretic Response

**Figure 6:**
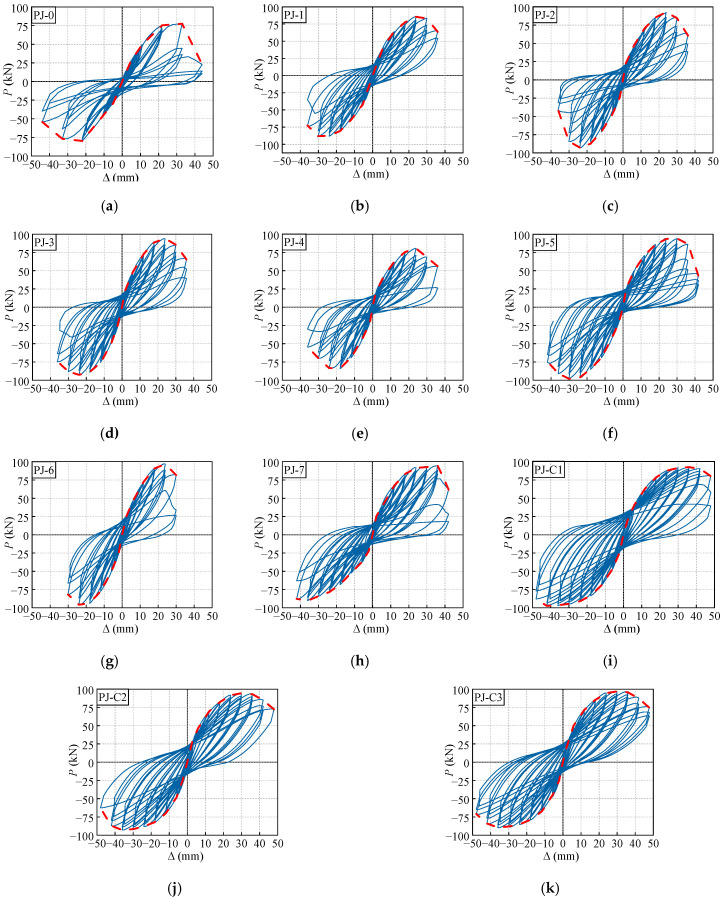
Hysteresis and skeleton curves of the specimens. (**a**) PJ-0. (**b**) PJ-1. (**c**) PJ-2. (**d**) PJ-3. (**e**) PJ-4. (**f**) PJ-5. (**g**) PJ-6. (**h**) PJ-7. (**i**) PJ-C1. (**j**) PJ-C2. (**k**) PJ-C3.

Figure 6 plots the hysteretic curves between the vertical load on the frame beam end *P* and the corresponding vertical displacement Δ. All hysteretic curves are characterized by the significant pinching effect and residual deformation, the evolution of the generic hysteretic curves is described as follows.

Before the specimens cracking, the hysteretic curves basically cycle linearly with a slight residual deformation. When the obvious crossed oblique cracks appearing in the joint core area, the hysteretic curves gradually progress into the elasto-plastic stage, in which stage, the growth rate of load is increasingly slower than the displacement, the pinching phenomenon is detected and becomes more pronounced with the increasing displacement. After the peak bearing capacity is reached, since the bond failure between the joint concrete and STC, the growth rate of load decreases drastically. Correspondingly, the apparent residual deformation and pinching effect is observed from the hysteretic loops, which indicates the significant degradation in strength and stiffness of the joints.

Additionally, as illustrated in Figure 6, the hysteretic curve of the specimen PJ-0 exhibits a premature pinching phenomenon and short yielding platform. In contrast, the hysteretic loops of the specimens PJ-1, PJ-2 and PJ-3 are relatively plump and show a fusiform shape, indicating that the seismic performance can be significantly enhanced after strengthening with STC. Meanwhile, the improvement in the stirrup ratio of the joint, reinforcement ratio of the frame beam or the decrease of axial compression ratio can also enlarge the area surrounded by the hysteretic loops. The CFRP strips spacing has no significant impact on the hysteretic behavior, mainly because the PFCC column is basically unimpaired when the specimens are damaged. This is in accordance with the results proposed by Ref. [45].

The *P*-Δ skeleton curves for each specimen are also presented in Figure 6, which are generated by sequentially connecting the peak points of the first hysteretic loop for each displacement loading, involving the seismic performance indicators such as bearing capacity and deformability, etc. Obviously, each specimen undergoes a prominent elastic stage, elasto-plastic stage, plastic hardening stage, and descending stage. The trajectories of all specimens exhibit slight diversity in the initial stage, indicating the impact of various structural parameters on the seismic performance is mainly reflected in the elasto-plastic stage and beyond.

**Figure 7:**
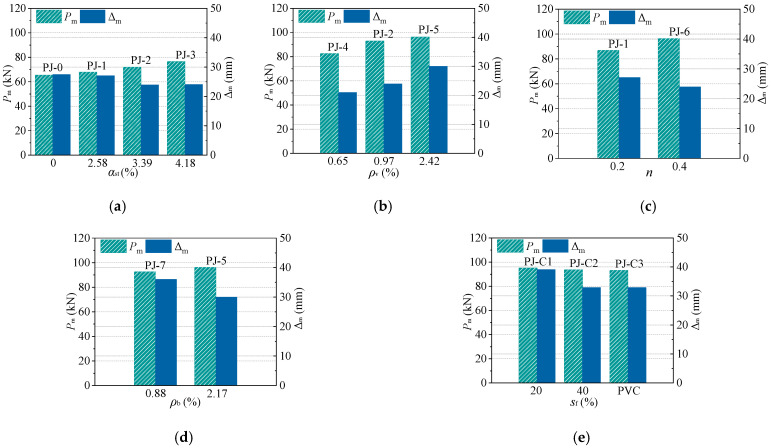
Effects of design parameters on the peak bearing capacity and deformation. (**a**) Steel ratio of STC. (**b**) Stirrup ratio of the joint. (**c**) Axial compression ratio. (**d**) Reinforcement ratio of the frame beam. (**e**) CFRP strips spacing.

For an intuitive discussion of the test results, the peak loads and displacements (The average values of the positive and negative directions of the points) are plotted in Figure 7. The peak bearing capacity of the specimens PJ-1, PJ-2, and PJ-3 increase by 10.6%, 18.2%, and 18.7% contrasting with that of the reference specimen PJ-0, respectively. This is because the STC can deliver a confinement on the encased concrete and participate in bearing the shear forces. Similar reason results in a higher peak bear capacity of 3.5% and 16.7% for the specimen PJ-5 compared with the specimens PJ-4 and PJ-2, respectively. Additionally, it is worth noting that when the steel ratio of STC exceeds 3.39%, the peak bearing capacity does not increase significantly as the steel ratio of STC increases. This is because, a certain bond failure is occurred between the joint concrete and the STC before the peak bearing capacity is reached. With the improvement in axial compression ratio, the compressive region of the joint concrete is enlarged, which is the reason for the 10.7% increase in peak bearing capacity of the specimen PJ-6 compared to the specimen PJ-1.

Moreover, it is observed that the peak displacements of the specimens are principally determined by the stirrup ratio of the joint and the reinforcement ratio of the frame beam. With the improvement in stirrup ratio of the joint, the development of the core concrete cracks is further confined, and the joints present a stronger deformability. Meanwhile, the plastic hinge on the frame beam root is more prone to adequately develop with a smaller reinforcement ratio of the frame beam, the deformability of the joints can also be enhanced.

### 3.3. Ductility

**Figure 8:**
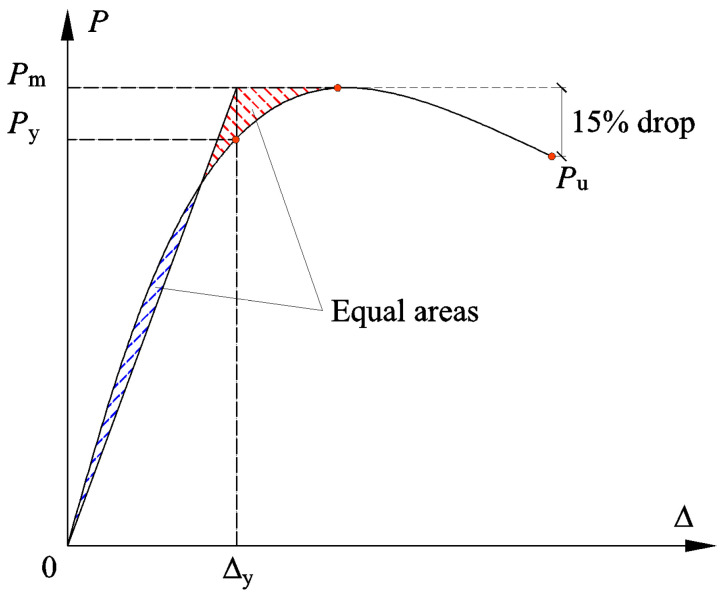
Illustration of energy equivalence method.

**Figure 9:**
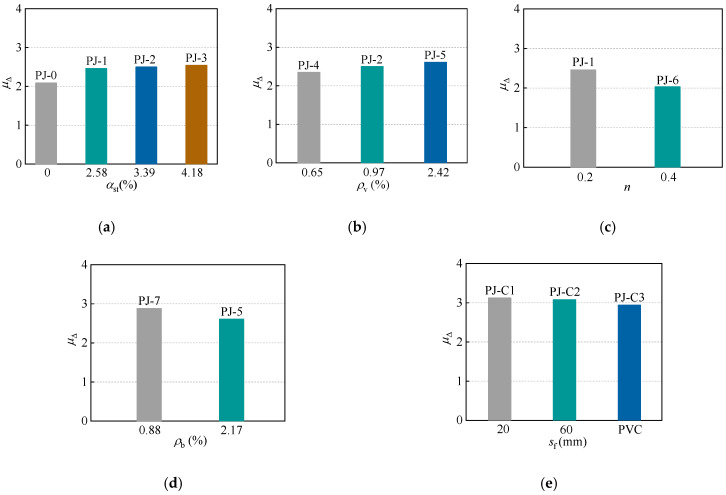
Effects of design parameters on the ductility coefficients. (**a**) Steel ratio of STC. (**b**) Stirrup ratio of the joint. (**c**) Axial compression ratio. (**d**) Reinforcement ratio of the frame beam. (**e**) CFRP strips spacing.

The ductility coefficient is a crucial indicator to quantify the deformability of the RC structures in seismic design, which is defined as the ratio of the ultimate displacement $\Delta_{u}$ to the yield displacement $\Delta_{y}$. Based on the skeleton curves established above, $\Delta_{u}$ is defined as the point corresponding to a 15% strength degradation from the peak point. $\Delta_{y}$ is determined by the energy equivalence method [44], as illustrated in Figure 8. The ductility coefficients of each specimen are summarized in Figure 9.

The ductility coefficients of the specimens PJ-0, PJ-1, PJ-2 and PJ-3 are 2.09, 2.46, 2.50 and 2.54, respectively. The ductility coefficient of the specimen PJ-3 is only slightly higher than that of the specimen PJ-1 attribute to the above-mentioned bond failure. Therefore, corresponding methods should be developed to avoid this phenomenon, such as welding ring bars or shear studs on the outer wall of STC. Alternatively, bonding (or bolting, etc.) CFRP profiles on the surface of the joint area as additional shear reinforcement is an option worth investigating [46,47,48,49,50]. However, the improvement is considerable compared to the ordinary joint. 

Additionally, as the stirrup ratio of the joint increases, the confinement on the core concrete at the periphery of the STC is enhanced, which can effectively inhibit the extension of the joint concrete cracks, resulting in a stronger ductility of the specimens. The ductility coefficient of the specimen PJ-5 with a higher reinforcement ratio of the frame beam is 10.3% less than that of the specimen PJ-7. This may due to the higher reinforcement ratio inhibiting the generation of the plastic hinge. It is worth mentioning that, although the specimen PJ-6 exhibits a considerable peak bearing capacity in the above description, its ductility is the lowest among the specimens, this is because the increasing axial compression ratio will further accelerate the development of concrete cracks. 

Furthermore, the ductility coefficient of each specimen ranges from 2.03 to 3.12, which satisfies the requirement that the ductility coefficient of RC structures is not less than 2 [44].

### 3.4. Strength Degradation

Since the cyclic loading is usually accompanied by a reduction in the bearing capacity for the identical displacement amplitude, the strength degradation coefficient $\lambda_{j}^{i}$ is utilized to estimate the degree of degradation, as shown in Equation (1).

(1) $$\lambda_{j}^{i}=P_{j}^{i}/P_{j}^{1}$$

where, $P_{j}^{i}$ and $P_{j}^{1}$ denote the peak load at the ith cycle and first cycle for the displacement amplitude level j, respectively.

**Figure 10:**
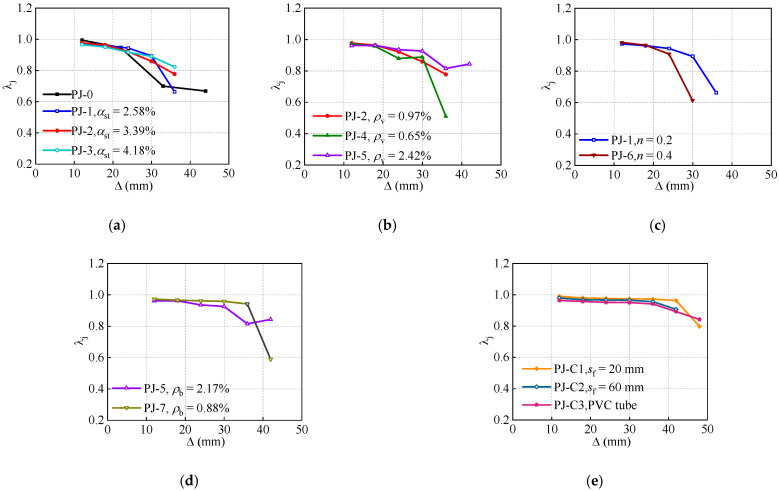
Effects of design parameters on the strength degradation coefficient. (**a**) Steel ratio of STC. (**b**) Stirrup ratio of the joint. (**c**) Axial compression ratio. (**d**) Reinforcement ratio of the frame beam. (**e**) CFRP strips spacing.

To simplify the calculation, the strength degradation coefficient at the second cyclic loading is adopted, as depicted in Figure 10. All specimens exhibit similar strength degradation coefficients in the initial stage, and the effect of each structural parameter on the stiffness degradation commences in the descending stage.

In the specimens with the steel ratio of STC and stirrup ratio of the joint as the comparison parameters, the specimens PJ-3 and PJ-5 show the gentlest descending stage. Manifesting that the improvement in the steel ratio of STC and stirrup ratio of the joint are relatively effective methods to strengthen the joints. Significantly, the turning point of the specimen PJ-1 is earlier than that of the specimen PJ-6, which suggests that the improvement in axial compression ratio will accelerate the strength degradation. The strength degradation coefficient of the specimen PJ-7 is maintained above 0.8, indicating the joints with lower reinforcement ratio of the frame beam can perform a better stability of the bearing capacity.

### 3.5. Stiffness Degradation

Stiffness is often described as the ability of the RC structures to resist the deformation. The loop stiffness $K_{j}$ is introduced to characterize the stiffness degradation of the joints, which is defined as Equation (2).

(2) $$K_{j}=\sum_{i=1}^{n}P_{j}^{i}/\sum_{i=1}^{n}\Delta_{j}^{i}$$

where, $\Delta_{j}^{i}$ denotes the peak displacement at the ith cycle for the displacement amplitude level j.

**Figure 11:**
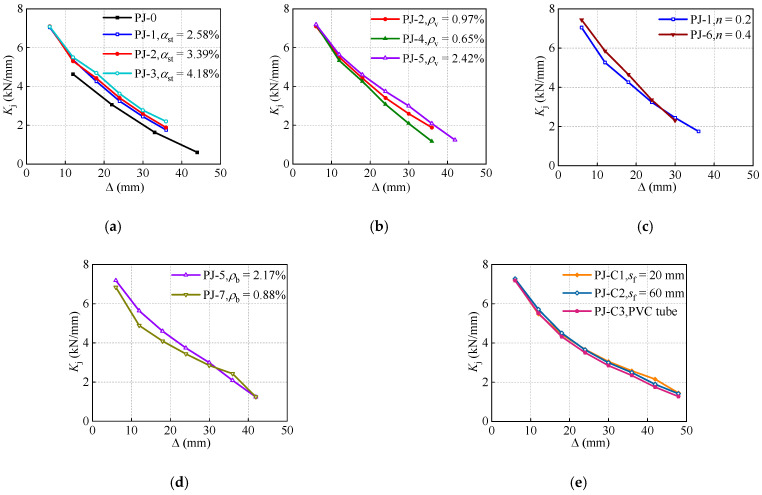
Effects of design parameters on the loop stiffness. (**a**) Steel ratio of STC. (**b**) Stirrup ratio of the joint. (**c**) Axial compression ratio. (**d**) Reinforcement ratio of the frame beam. (**e**) CFRP strips spacing.

As shown in Figure 11, a similar law to the strength degradation is revealed in the stiffness degradation, presenting two distinct rates of degradation. After the turning point, the specimens PJ-3 and PJ-5 with a higher steel ratio of STC and stirrup ratio of the joint present a more stable stiffness degradation than that of the specimens PJ-0, PJ-1, PJ-2 and PJ-4. This behavior further demonstrates the effectiveness of the STC and joint stirrups in enhancing the seismic performance of the proposed joint. In contrast with the strength degradation, the specimen PJ-1 exhibits a slightly lower initial stiffness compared with the specimen PJ-6. However, as the loading proceeds, the specimen PJ-6 presents a more severe tendency of stiffness degradation due to the accelerated cracks development with the axial compression ratio increases.

### 3.6. Energy Dissipation

**Figure 12:**
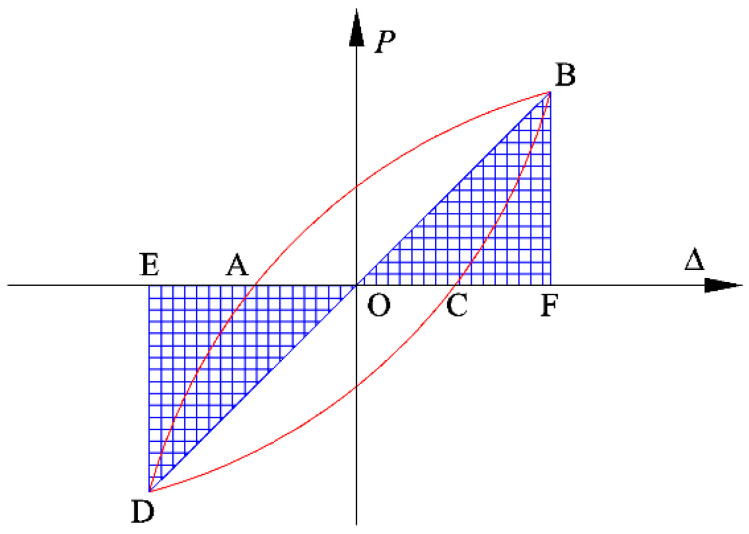
Typical hysteretic loop.

The equivalent viscous damping ratio $h_{e}$ is generally recognized as an indicator to evaluate the magnitude of the energy dissipation capacity. As shown in Figure 12, the $h_{e}$ is defined as Equation (3).

(3) $$h_{e}=\frac{1}{2\pi }\times \frac{S_{1}}{S_{2}+S_{3}}$$

where, $S_{1}$, $S_{2}$ and $S_{3}$ are the areas enveloped by the hysteretic loop ABCDA, triangle OBF and triangle ODE, respectively.

**Figure 13:**
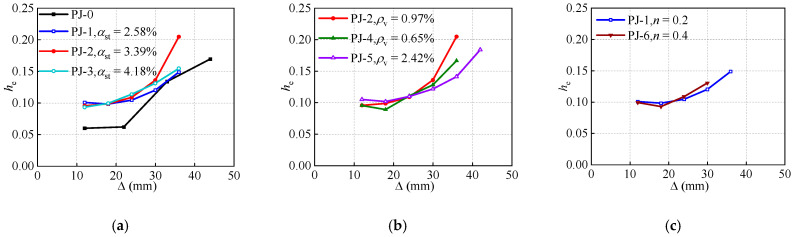

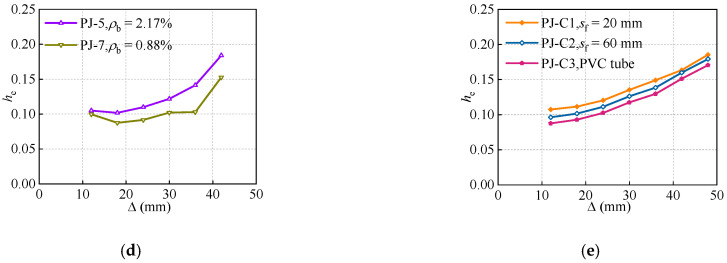
Effects of design parameters on the equivalent viscous damping ratio. (**a**) Steel ratio of STC. (**b**) Stirrup ratio of the joint. (**c**) Axial compression ratio. (**d**) Reinforcement ratio of the frame beam. (**e**) CFRP strips spacing.

The development of the $h_{e}-\Delta$ curves is presented in Figure 13. In combination with the skeleton curves, the $h_{e}-\Delta$ curves are classified into the stable stage and ascending stage. It can be observed that the $h_{e}$ of the joints with STC are higher than that of the ordinary joint throughout the loading process. However, owing to the bond failure between the joint concrete and STC, the STC does not yield completely, resulting in the effect of the steel ratio of STC on the $h_{e}$ is not noticeable. 

In addition, the impact of the stirrup ratio of the joint on the $h_{e}$ is revealed in the ascending stage, i.e., the $h_{e}$ increases with the improvement in the stirrup ratio of the joint. Interestingly, the $h_{e}$ of the specimen PJ-5 is smaller than that of the specimens PJ-2 and PJ-4 after the displacement reaches 36 mm. This is due to the fact that the confining effect from the joint stirrups of the specimen PJ-5 is not entirely developed. On the other hand, the specimen PJ-5 exhibits a more favorable capability of cumulative energy dissipation. As the axial compression ratio increases, the $h_{e}-\Delta$ curves present a faster growth rate. This is mainly because, the lateral deformation of the joint concrete expands correspondingly with the increasing axial compression ratio. However, it also induces the earlier damage of the specimens. As expected, the CFRP strips spacing exhibits a slight impact on the stiffness degradation, nevertheless, as described in the Ref. [51], the efficacy of the CFRP strips in the joints with STC can probably be found in the high axial compression ratio specimens. In addition, the specimen PJ-5 presents a relatively excellent energy dissipation capacity, especially in the ascending stage, where its $h_{e}$ is 20.9% to 38.5% higher than that of the specimen PJ-7, which may attribute to the increased flexural stiffness of the frame beam.

## 4. Numerical Modelling

OpenSees is a finite element software for simulating the seismic response of engineering structures, which shows the advantages of short computation time-consumption and high solution efficiency. The skeleton curves of the exterior joint with STC under low cyclic loading is numerically simulated by the fiber beam-column element in the OpenSees, and the further parametric analysis of the joints is performed.

### 4.1. Constitutive Model of the Materials

(1) Concrete

**Figure 14:**
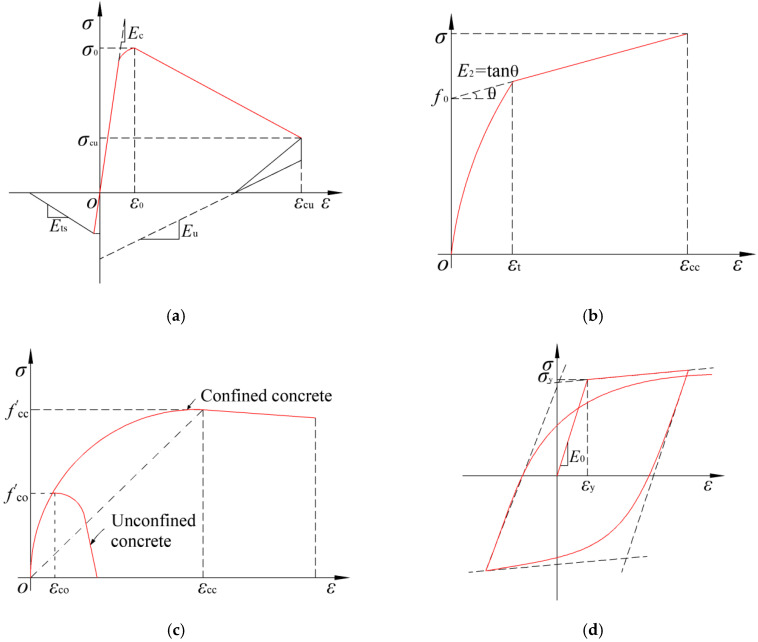
Material models. (**a**) Concrete02 model. (**b**) PVC-FRP confined concrete. (**c**) Joint core concrete. (**d**) Steel02 model.

The ‘Concrete02′ and ‘Concrete03′ models are commonly adopted in OpenSees to simulate the concrete under seismic action since they can incorporate the stiffness degradation and hysteretic energy dissipation of the concrete during the loading and unloading. Nevertheless, in view of the time consumption of the concrete03 model, model concrete02 is introduced in the analysis models of the sandwiched concrete and ordinary concrete. The stress–strain relationship of the proposed model is illustrated in Figure 14a.

The properties of PVC-FRP confined concrete are defined according to the constitutive model proposed by Fang et al. [13], as shown in Figure 14b. For the joint core concrete, considering it is not only confined by the STC, the confinement produced by the peripheral stirrups confine concrete is also considerable, the steel tube confined concrete model proposed by Han [52] is not applicable for the present test. Thus, for instead, the modified confined concrete constitutive model proposed by Mander [53] is selected, the specific modification is to adjust the improvement factor *η* from 5 to 5.5 to incorporate the confining effect of the STC, as shown in Figure 14c.

(2) Steel

The simulated results of the ‘steel02’ model are in good agreement with the cyclic loading tests on the steel materials. Meanwhile, the ‘steel02′ model can fully reveal the kinematic hardening effect and nonlinear combined isotropic. Thus, the ‘steel02′ model proposed by Filippou et al. [54] is utilized for the reinforcements and steel tubes, as demonstrated in Figure 14d.

### 4.2. Fiber Beam-Column Element

**Figure 15:**
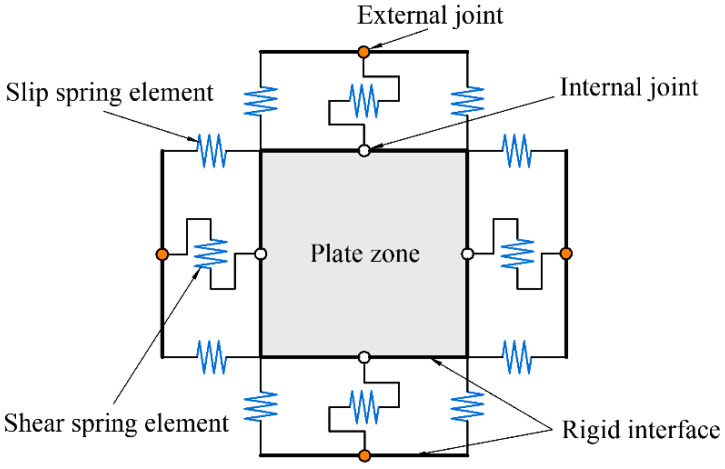
Analytical model of the joint specimens.

The joint core area is modelled with fiber cross sections, as presented in Figure 15. The beam-column element model is composed of four internal joints and four external joints, and all internal and external joints are located at a rigid interface. Each external joint has one degree of freedom for rotation and two degrees of freedom for translation to ensure the compatibility of the frame beam and column elements. Between the two rigid interfaces, eight zero-length slip spring elements are developed to consider the degradation of the stiffness and strength owing to the bond slip between the longitudinal reinforcements and concrete. The degradation of the shear force transmission capacity at the interface around the joints is simulated by four zero-length shear spring elements. Additionally, the degradation of strength and stiffness caused by the joint shear failure is simulated by the plate zone.

### 4.3. Modelling of the Shear Behavior in the Joint Core Area

To accurately model the joint core area subjected to low cyclic loading, the shear behavior of the panel zone should be specified. The diagonal strut model [55,56] is suggested to determine the parameters of the shear stress-strain skeleton curves of the plate zone, the height of the diagonal strut $a_{s}$ is regarded to be constant during the loading process, and the empirical formula $a_{s}=\sqrt{a_{c}^{2}+a_{b}^{2}}$ is selected to determine its value. Therefore, the geometry of the diagonal strut is calculated as follows.

(4) $$\theta =\arctan(h_{b}/h_{c})$$

(5) $$a_{b}=(0.121+3.21\rho_{v})h_{b}$$

(6) $$a_{c}=(0.37+0.53n+0.04\xi_{\mathrm{ef}}-1.44\rho_{c})h_{c}$$

where, θ denote the inclination of the diagonal strut. $\xi_{\mathrm{ef}}$ is the confining effect coefficient of the CFRP strips [57]. $h_{b}$ and $h_{c}$ are the section height of the frame beam and the PFCC column, respectively. $a_{b}$ and $a_{c}$ are the height of compression zone of the frame beam and the PFCC column, respectively. 

Meanwhile, owing to the panel zone consists of the joint stirrups, sandwiched concrete and steel tube confined concrete, its shear behavior is distinct from the CFST structures. On the basis of the analytic results in Ref. [36], to completely reveal the doubly confining effect of the joint stirrups and steel tube on the core concrete, the modified confined concrete constitutive model implemented by Mander [53] is adopted, and the shear stress $\tau_{j}$ and shear deformation $\gamma_{j}$ of the panel zone are calculated as follows.

(7) $$\tau_{j}=\frac{f_{\mathrm{cj}}A_{\mathrm{str}}\cos\theta }{A_{j}}=\frac{f_{\mathrm{cj}}a_{s}b_{s}\cos\theta }{A_{j}}$$

(8) $$\gamma_{j}=\frac{\varepsilon_{\mathrm{cj}}\sqrt{h_{b}^{2}+h_{c}^{2}}}{h_{b}\cos\theta }=\frac{\varepsilon_{\mathrm{cj}}}{\sin\theta \cos\theta }$$

where, $A_{\mathrm{str}}$, $a_{s}$, $b_{s}$, $f_{\mathrm{cj}}$ and $\varepsilon_{\mathrm{cj}}$ are the area, width, height, axial compressive stress and strain of the diagonal strut, respectively. $A_{j}$ denote the total area of the joint core area.

### 4.4. Validation of the FE Model

**Figure 16:**
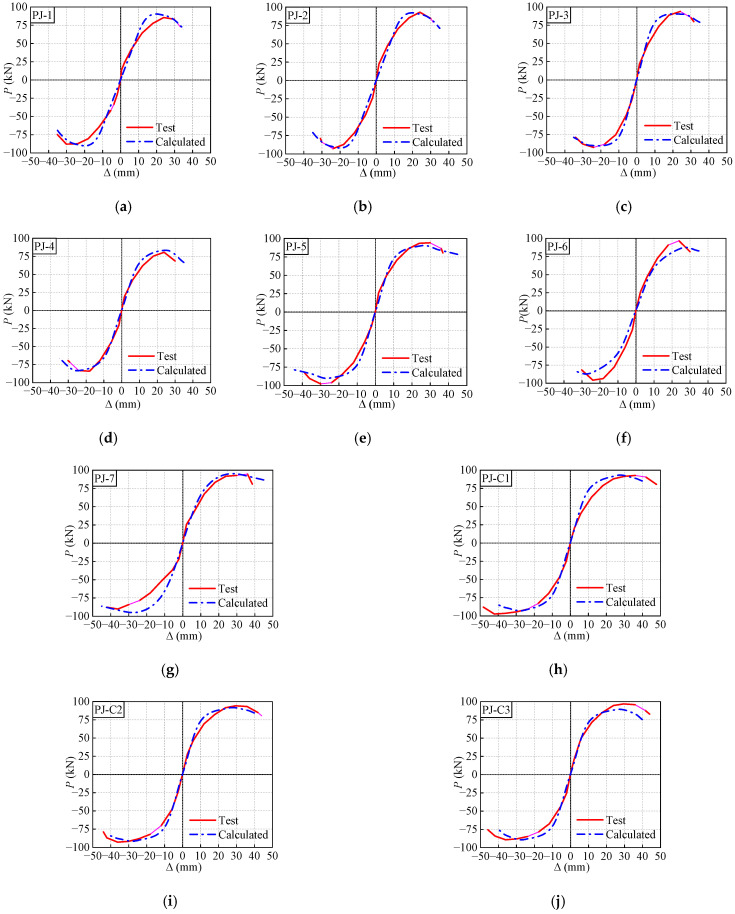
Comparison between the test and calculated skeleton curves. (**a**) PJ-1. (**b**) PJ-2. (**c**) PJ-3. (**d**) PJ-4. (**e**) PJ-5. (**f**) PJ-6. (**g**) PJ-7. (**h**) PJ-C1. (**i**) PJ-C2. (**j**) PJ-C3.

Through the above modeling steps, the numerical *P*-Δ skeleton curves are developed, as illustrated in Figure 16. It can be found that the skeleton curves obtained numerically and experimentally present highly overlapped, which verifies the accuracy of the established FE model.

### 4.5. Parameter Analysis

**Figure 17:**
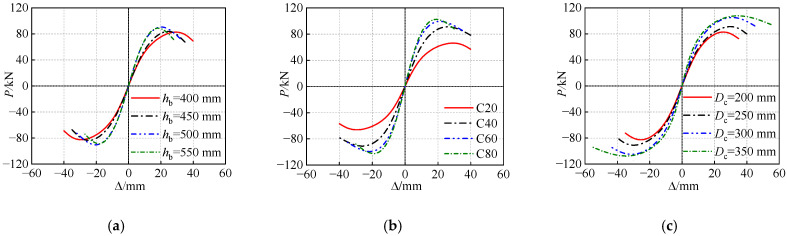
Effects of various parameters on the numerical skeleton curves. (**a**) Joint heigh. (**b**) Joint concrete strength. (**c**) Joint diameter.

On the basis of the validated FE model, additional parameters which may influence the seismic behavior of the joints are further analyzed, for the purposes of improving the seismic design of this composite structure. The *P*-Δ skeleton curves with the various parameters are illustrated in Figure 17. The specimen designs for parameter analysis are the same as that of the specimen PJ-2 unless otherwise stated.

As illustrated in Figure 17a, with the joint heigh varies from 400 mm to 550 mm with the increment of 50 mm, the bearing capacity of the simulated specimens does not exhibit distinct differences (Maximum variation is 8%). However, since the height of the frame beam is consistent with the joint, and the larger dimension of the frame beam is disadvantageous for the plastic hinge development, which result in the seismic performances including the deformability and ductility are weakened at different levels as the joint height increases. Additionally, it is worth noting that as the joint height increases from 500 mm to 550 mm, the peak bearing capacity is not improved while the reduction in the peak displacement is observed. Hence, it can be concluded that the joint height below 500 mm appear to be an optimum solution for the proposed joint.

As illustrated in Figure 17b, increasing the joint concrete strength from C20 to C80 improves the corresponding peak bearing capacity by 37.5%, 50.2% and 54.7%, respectively. However, as the concrete strength exceeds C60, the improvement in bearing capacity is negligible, while the stiffness degradation is accelerated. This may be attributed to the fact that concrete with higher strength is more prone to brittle failure. This observation suggests that the joint concrete with strength above C60 may not provide better seismic behavior for the proposed joint.

As illustrated in Figure 17c, the skeleton curves present a consistent trend with increasing joint diameter, i.e., both the bearing capacity and deformability are enhanced accordingly. Noticeably, increasing the joint diameter from 300 mm to 350 mm, the bearing capacity is hardly changed because the joint core is undestroyed, and the strength is not fully utilized when the specimen failure, while the ultimate displacement has a considerable development of 22.2%. Considering the fact that the excessive size of the joint is not conducive to construction and architectural aesthetics, the joint diameter of the proposed joint should not exceed 300 mm.

## 5. Conclusions

Seismic behaviors were investigated on the RC beam to PFCC column exterior joint with STC through the experiment and numerical simulation in this study. The following conclusions can be obtained:

1. The final failure mode exhibited by all specimens is a joint shear failure. The increase in the stirrup ratio of the joint or the decline in the axial compression ratio can effectively confine the crack development of the joint concrete.
2. The proposed joint with STC presents a superior seismic performance when compared with the ordinary joint. However, the strength of STC cannot be completely utilized owing to the premature bond failure between the joint concrete and STC.
3. By increasing the steel ratio of STC or the stirrup ratio of the joint, the pinching effect of the hysteretic curves is controlled, the ductility and energy dissipation capacity are enhanced, and the strength degradation and stiffness degradation are delayed, while the increase in the reinforcement ratio of the frame beam and axial compression ratio presents the opposite effects.
4. The higher stirrup ratio of the joint, axial compression ratio and steel ratio of STC develops a better contribution to the peak bearing capacity. Meanwhile, the peak displacement of the specimens observably increases as the stirrup ratio of the joint increases or the reinforcement ratio of the frame beam decreases.
5. The numerical model of the joint is established by OpenSees, and it is verified that the developed model can well simulate the skeleton curves of the proposed joint.
6. The parametric numerical analysis indicates that the peak bearing capacity increases with the concrete strength, height or diameter of the joint increase, while the increase in the height of the joint can also result in the reduction of the peak displacement. Considering the bearing capacity, deformability and construction convenience, it is recommended that the joint height be below 500 mm, the joint concrete strength be below C60 and the joint diameter not exceed 300 mm.

## Figures and Tables

**Table 1:** The detailed parameters of the specimens.

No.	*d*_sc_ × *t*_sc_ (mm)	$\alpha_{\mathrm{st}}$ (%)	$\rho_{b}$ (%)	n	$\rho_{v}$ (%)	$S_{f}$ (mm)	$\rho_{c}$ (%)
PJ-0	-	-	2.17% (4C20)	0.2	0.97% (A6.5@100)	40	4.86% (6C18)
PJ-1	76 × 3	2.58%	2.17% (4C20)	0.2	0.97% (A6.5@100)	40	4.86% (6C18)
PJ-2	76 × 4	3.39%	2.17% (4C20)	0.2	0.97% (A6.5@100)	40	4.86% (6C18)
PJ-3	76 × 5	4.18%	2.17% (4C20)	0.2	0.97% (A6.5@100)	40	4.86% (6C18)
PJ-4	76 × 4	3.39%	2.17% (4C20)	0.2	0.65% (A6.5@150)	40	4.86% (6C18)
PJ-5	76 × 4	3.39%	2.17% (4C20)	0.2	2.42% (A6.5@40)	40	4.86% (6C18)
PJ-6	76 × 3	2.58%	2.17% (4C20)	0.4	0.97% (A6.5@100)	40	4.86% (6C18)
PJ-7	76 × 4	3.39%	0.88% (2C18)	0.2	2.42% (A6.5@40)	40	4.86% (6C18)
PJ-C1	76 × 5	4.18%	2.17% (4C20)	0.2	3.00% (A8@50)	20	1.50% (6C10)
PJ-C2	76 × 5	4.18%	2.17% (4C20)	0.2	3.00% (A8@50)	60	1.50% (6C10)
PJ-C3	76 × 5	4.18%	2.17% (4C20)	0.2	3.00% (A8@50)	-	1.50% (6C10)

Note: *d*_sc_ and *t*_sc_ denote the external diameter and thickness of STC, respectively. *A*_st_ = *A*_st_/*A*_j_, *A*_st_ and *A*_j_ are the section area of the STC and joint core, respectively. *ρ*_c_ represents the reinforcement ratio of the PFCC column. *n* = *N*_0_/*Af*_c_, *N*_0_ is the axial compression on the column head. *A* is the section area of the column. *F*_c_ is the axial compressive strength of the concrete. “-” means no STC or CFRP strips.

**Table 2:** Mechanical index of the steel, PVC tubes, FRP strips and reinforcements.

Materials		Thickness/ Diameter (mm)	Tensile Strength (Mpa)	Yield Strength (Mpa)	Elastic Modulus (Mpa)
Steel	Steel tubes	3	461	314	2.01 × 10^5^
		4	425	301	1.99 × 10^5^
		5	382	285	1.97 × 10^5^
	Stirrups (HRB300)	6.5	543	349	1.98 × 10^5^
		8	441	306	2.00 × 10^5^
		10	432	313	1.97 × 10^5^
	Longitudinal reinforcements (HRB400)	10	620	451	1.95 × 10^5^
		14	703	486	1.98 × 10^5^
		20	656	443	2.01 × 10^5^
PVC tube		7.8	69.6	-	2.59 × 10^3^
CFRP strip		0.111	3795.6	-	2.74 × 10^5^

## Data Availability

The data presented in this study are available on request from the corresponding author.
